# Dietary Influences on Gut Microbiota and Their Role in Metabolic Dysfunction-Associated Steatotic Liver Disease (MASLD)

**DOI:** 10.3390/nu17010143

**Published:** 2024-12-31

**Authors:** Sevag Hamamah, Oana C. Iatcu, Mihai Covasa

**Affiliations:** 1 Department of Internal Medicine, Scripps Mercy Hospital, San Diego, CA 92103, USA; hamamah.sevag@scrippshealth.org; 2 Department of Biomedical Sciences, College of Medicine and Biological Science, University of Suceava, 720229 Suceava, Romania; oana.iatcu@usm.ro

**Keywords:** liver disease, gut bacteria, Western diet, Mediterranean diet, ketogenic diet, intermittent fasting

## Abstract

Metabolic dysfunction-associated steatotic liver disease (MASLD) is a major contributor to liver-related morbidity, cardiovascular disease, and metabolic complications. Lifestyle interventions, including diet and exercise, are first line in treating MASLD. Dietary approaches such as the low-glycemic-index Mediterranean diet, the ketogenic diet, intermittent fasting, and high fiber diets have demonstrated potential in addressing the metabolic dysfunction underlying this condition. The development and progression of MASLD are closely associated with taxonomic shifts in gut microbial communities, a relationship well-documented in the literature. Given the importance of diet as a primary treatment for MASLD, it is important to understand how gut microbiota and their metabolic byproducts mediate favorable outcomes induced by healthy dietary patterns. Conversely, microbiota changes conferred by unhealthy dietary patterns such as the Western diet may induce dysbiosis and influence steatotic liver disease through promoting hepatic inflammation, up-regulating lipogenesis, dysregulating bile acid metabolism, increasing insulin resistance, and causing oxidative damage in hepatocytes. Although emerging evidence has identified links between diet, microbiota, and development of MASLD, significant gaps remain in understanding specific microbial roles, metabolite pathways, host interactions, and causal relationships. Therefore, this review aims to provide mechanistic insights into the role of microbiota-mediated processes through the analysis of both healthy and unhealthy dietary patterns and their contribution to MASLD pathophysiology. By better elucidating the interplay between dietary nutrients, microbiota-mediated processes, and the onset and progression of steatotic liver disease, this work aims to identify new opportunities for targeted dietary interventions to treat MASLD efficiently.

## 1. Introduction

Metabolic dysfunction-associated steatotic liver disease (MASLD), formerly known as non-alcoholic fatty liver disease (NAFLD) or non-alcoholic steatohepatitis (NASH), remains a significant contributor to the growing global burden of liver-disease-related mortality [1]. Globally, viral hepatitis remains the leading cause of cirrhosis; however, with widespread vaccination efforts, advances in hepatitis treatment and increased prevalence of obesity, MASLD is steadily increasing across several regions of the world including Europe, the Middle East, and the Americas [1,2]. While not all cases of MASLD progress to cirrhosis, it is estimated that 38% of all adults and 7–14% of adolescents meet the diagnostic criteria for MASLD, with projections indicating prevalence could surpass 55% in adults by 2040 [3]. In certain populations, such as women in the United States, MASLD has become the leading cause for liver transplantation [3]. The implications of MASLD extend beyond the liver, contributing to adverse effects on cardiovascular health, chronic kidney disease, and cancers [2]. Given its mortality, morbidity, and economic burden, substantial research efforts are focused on developing novel pharmacological treatments for its prevention and management [2]. As such, a deeper understanding of MASLD pathophysiology is critical to guiding upstream primary prevention strategies and fostering the development of innovative treatment modalities that extend beyond pharmacological interventions.

Likewise, it is important to address the transition in terminology and diagnostic criteria from NAFLD to MASLD aimed at improving diagnostic accuracy and establishing a global consensus [4]. The new framework provides clearer differentiation between MASLD and other liver diseases such as alcohol-related liver disease or viral hepatitis. The term “MASLD” underscores the complex interplay of metabolic and environmental factors driving the condition, while reducing the stigma associated with the term “non-alcoholic” [5]. The updated diagnostic criteria for MASLD in adults, as defined by the American Association for the study of Liver Disease (AASLD), require the presence of liver steatosis concurrently with one of five cardiometabolic risk factors: (1) Body Mass Index > 25; (2) diagnosed hypertension, dyslipidemia, or type 2 diabetes mellitus (T2DM); (3) treatment for T2DM; (4) elevated plasma triglycerides > 1.7 mmol/L or lipid-lowering treatment; (5) low-plasma high-density lipoprotein (HDL) < 1 mmol/L in males and <1.3 mmol/L in females [4,6]. Screening for other major contributors such as alcohol and hepatitis B/C should also be conducted to rule out superimposed chronic liver injury [6]. Patients who meet criteria for MASLD but also and have increased alcohol intake are diagnosed under the new terminology of MetALD [6].

Currently, lifestyle modifications remain the first-line approach for primary prevention and management of MASLD pathogenesis [7]. Among these, dietary habits, in conjunction with exercise, play an important factor in achieving sustainable weight loss, which is linked to significant improvements in insulin resistance and metabolic parameters associated with steatotic liver disease [8]. A weight loss of approximately 7–10% of total body weight has been shown to markedly reduce hepatic fat accumulation, inflammation, and fibrosis [9]. The mechanisms through which dietary interventions influence MASLD pathophysiology are multifaceted, including but not limited to reducing de novo lipogenesis, enhancing fatty acid oxidation, mitigating inflammation, reducing oxidative stress, improving insulin sensitivity, ameliorating autophagy, and optimizing gut microbial composition [10,11,12,13,14].

Emerging evidence underscores the critical role gut microbiota play as an intermediary between nutrition and the pathogenesis or prevention of MASLD [15]. Gut microbiota also play a key role in individual risk factors for MASLD including dyslipidemia, T2DM, and hypertension [16,17,18,19]. Reduced microbial diversity and states of dysbiosis are a hallmark characteristic of MASLD development and associated cardiometabolic risk factors [20]. Important markers of dysbiosis, such as increased Firmicutes/Bacteroidetes ratio, Proteobacteria abundance, and overgrowth of the *Enterobacteriaceae* microbial family, promote inflammation, leading to “leaky gut” and systemic translocation of endotoxins to various organs including the liver [21,22]. Interestingly, many of the pathophysiologic mechanisms that drive pathogenic effects of unhealthy dietary intake on MASLD are driven by microbiota and their related metabolites [23]. For example, consumption of a Western-based diet enhances liver-mediated fatty acid synthesis and cholesterol uptake through disruption of gut microbiota [24]. Further, unfavorable dietary interventions induce hepatic inflammation via dysregulation of T-regulatory cells and up-regulation of pro-inflammatory pathways [25,26]. Conversely, incorporating a beneficial diet, such as the Mediterranean diet, has the opposite effect through enhancing gut microbial composition and improvement of physiological processes reducing hepatic inflammation and adiposity [27]. Microbial metabolic byproducts derived from dietary intake attenuate hepatic lipogenesis, inflammation, and oxidative stress [28]. Similarly, consumption of fruits/vegetables mitigates hepatic inflammation and strengthens the gut barrier via the microbiota metabolite butyrate [29].

Given the interrelationship between diet, gut microbiota composition, and metabolic disease, targeting gut microbiota through dietary interventions offers a promising strategy for the primary prevention of MASLD. This review first explores notable trends in gut microbiota associated with the pathophysiology of MASLD and related metabolic disorders. It then examines the mechanisms by which microbiota and their metabolites influence processes such as hepatic inflammation, lipogenesis, bile acid regulation, insulin resistance, and oxidative stress. Next, dietary interventions that lead to unfavorable taxonomical shifts in gut microbiota and the pathways through which these changes influence biological processes to induce steatotic liver changes are discussed. Finally, emerging evidence regarding the role of beneficial dietary interventions including the Mediterranean diet, ketogenic diet, intermittent fasting, and high-fiber diets in improving gut microbial composition and mitigating MASLD pathogenesis and progression is presented. Overall, this comprehensive review focuses on elucidating the complex interplay between diet, gut microbiota, and metabolic pathways in MASLD, highlighting the impact of both detrimental and beneficial dietary patterns on these processes. It addresses critical gaps in the understanding of the mechanistic links between dietary intervention, gut microbial composition, and their downstream effects on MASLD pathogenesis and progression. By deepening our understanding of how specific dietary patterns can be strategically leveraged to modulate gut microbiota, this knowledge aims to guide development of targeted nutritional strategies and personalized dietary recommendations to prevent and mitigate MASLD development.

## 2. Gut Microbiota Trends in MASLD and Related Pathophysiologic Mechanisms

The gut microbiota comprises the trillions (10^13^–10^14^) of microorganisms that reside in the human intestinal tract, playing a pivotal role in maintaining host health [30]. When microbial diversity is preserved, it supports essential physiologic processes such as energy harvesting, nutrient synthesis, immunomodulation, and promoting the integrity of the intestinal barrier [31]. Conversely, dysbiosis, or gut microbial imbalance, has detrimental effects human health through production of harmful metabolites, inflammation via endotoxin formation, and increased permeability of the intestinal barrier, leading to a “leaky gut” and negative systemic effects [32]. Dysbiosis significantly contributes to metabolic endotoxemia and development of chronic metabolic conditions including MASLD-associated risk factors like obesity, dyslipidemia, and T2DM [33]. Interestingly, both gut microbial composition and development of metabolic disorders, including MASLD, are influenced by similar extrinsic and intrinsic factors [34,35]. These include diet, exercise, age, environmental stressors, and medications, as well as epigenetics, immune status, and comorbid disease [35], underscoring the role of microbiota alterations as a potential driving factor in the development of metabolic disease. The gut and liver are intimately connected through the portal venous system, which transports significant amounts of blood and consequently microbial metabolites and endotoxins from the intestinal tract to the liver [36]. In states of dysbiosis and metabolic endotoxemia, microbial derivatives and toxic products that leak into the bloodstream are delivered to the liver, contributing to steatotic changes [37]. This intricate interrelationship between the gut and liver is commonly referred to as the “gut–liver axis” [38].

In recent years, the onset of hepatic steatosis has been associated with signature trends in gut microbial composition, starting with key taxonomical shifts in the main phyla, trickling down to important genera and species [39,40,41]. The human gut microbial composition consists of six main phyla, 90% comprised of Bacteroidetes and Firmicutes, while Proteobacteria, Fusobacteria, Verrucomicrobia, and Actinobacteria make up the remaining 10% [42]. In a cross-sectional study involving 37 patients with steatotic liver disease, Bacteroidetes, Firmicutes, and Proteobacteria emerged as the predominant phyla with an elevated Firmicutes/Bacteroidetes ratio strongly associated with hepatic steatosis and obesity [22]. Interestingly, of the three phyla, increased Proteobacteria was strongly correlated with hepatic fibrosis in patients with normal body mass indices [22], suggesting its role as an early marker of steatotic liver disease progression to cirrhosis [43]. Although Proteobacteria accounts for less than 5% of total gut microbial composition, their abnormal expansion is a hallmark of dysbiosis often disrupting microbial balance and precipitating metabolic dysfunction [44]. This increase in Proteobacteria has been attributed to an increase in pathogenic bacterial species such as *Escherichia* within the *Enterobacteriaceae* family [45]. At the genus level, *Bacteroides* predominates in individuals with hepatic steatosis, while *Prevotella* is more common in healthy populations [22,46]. These enterotypes, which categorize microbiota into distinct clusters based on relative abundance of specific microbes, are intricately linked to dietary responses as individuals with higher *Prevotella/Bacteroides* ratios experience greater weight loss in response to dietary, lifestyle, and disease-treatment interventions [46,47].

Recent meta-analyses have provided a further characterization of gut microbial signatures in patients with MASLD [40,48,49,50,51], revealing comparable results. In MASLD, α-diversity, a measure of microbial richness and distribution, showed significant reduction as assessed by the Shannon Index, a statistical measure of biodiversity in a specific habitat [51,52]. Lower α-diversity has been associated with worse health outcomes and serves as an important measure of microbiota health [53]. Similarly, β-diversity, comparing the difference in microbial composition between MASLD patients and healthy controls, reveals significant alterations linked with steatotic liver changes [51]. β-Diversity is another important microbial metric that compares similarity and differences between microbial samples or environments [54]. Importantly, anti-inflammatory species within the families *Rumminococcacae* and *Lachnospiraceae* are markedly reduced, whereas proinflammatory species from the *Desulfovibrionaceae* family are more prevalent, particularly in obese individuals with MASLD [48,55]. At the genus level, MASLD is associated with an increased relative abundances of proinflammatory *Escherichia*, *Streptococcus*, *Bilophila*, *Fusobacterium*, *Dorea*, and *Megaspheara.* Conversely, beneficial genera such as *Faecalibacterium*, *Coprococcus*, *Ruminococcus*, *Alistipes*, *Akkermansia*, *Blautia*, *Flavobacterium*, and *Coprobactera* are significantly decreased [40,48,49,50,51,56]. Certain microbial genera were associated with effects on metabolic serum markers. For example, *Dorea* overrepresented in MASLD and correlated with increased liver enzymes such as aspartate aminotransferase (AST) and alanine aminotransferase (ALT) [40]. Conversely, *Alistipes* decreased in steatotic liver disease and was negatively correlated to serum glucose, gamma-glutamyl transferase, and ALT [40].

Certain microbial genera and taxonomical alterations have been associated with progression of hepatic steatosis to cirrhosis [48,57,58]. In clinical practice, steatotic liver disease is staged from F0 to F4, based on the degree of fatty deposition, fibrosis, and scarring as assessed by elastography and confirmed by liver biopsy [59]. In a cross-sectional study of 60 patients with steatotic liver disease, individuals with significant fibrosis had higher proportions of *Bacteroides* compared to those without significant fibrosis [57]. Conversely, beneficial bacterial genera such as *Lactobacillus* and *Bifidobacterium* were notably reduced in patients with advanced fibrosis [57]. Similarly, species within the genera *Bacteroides*, *Dorea*, *Clostridium*, and *Streptococcus* were significantly associated with fibrotic liver changes in patients with biopsy-proven MASLD-induced cirrhosis [58]. Further, the *Enterobacteriaceae* family, including *Escherichia* and *Shigella*, had a sixfold increased association with significant fibrosis (F2–F4) compared to those in earlier stages F0/F1 [60,61].

To summarize, an overabundance of enteric, inflammatory, and pathogenic microbial genera including *Escherichia*, *Streptococcus*, *Shigella*, *Dorea*, *Fusobacterium*, and *Enterococcus* is strongly associated with the onset and fibrotic progression of steatotic liver disease. These bacterial genera play specific roles and influence key metabolite pathways, such as hepatic inflammation, lipogenesis, bile acid metabolism, and oxidative stress within hepatocytes, contributing to the pathophysiological changes observed in MASLD. Conversely, genera that are relatively reduced in individuals with steatotic liver disease such as *Lactobacillus*, *Akkermansia*, *Alistipes*, and *Eubacterium* modulate these same pathways to mitigate MASLD progression and prevent its onset. These intricate mechanisms, specific microbial roles, metabolite signaling pathways, and host interactions will be explored in detail in the following subsections. It is important to note, however, that these microbial genera are part of a highly dynamic and interdependent ecosystem, influencing host physiology both synergistically and antagonistically [62,63]. Therefore, the relative abundance or reduction of these genera is context-dependent, and their interactions are not binary. Rather, they involve networks of metabolite exchange signaling, immune modulation, and environmental influences, such as diet, which collectively influence steatotic liver changes [64].

### 2.1. Gut Microbes, Hepatic Inflammation, and Steatotic Liver Disease

**Figure 1:**
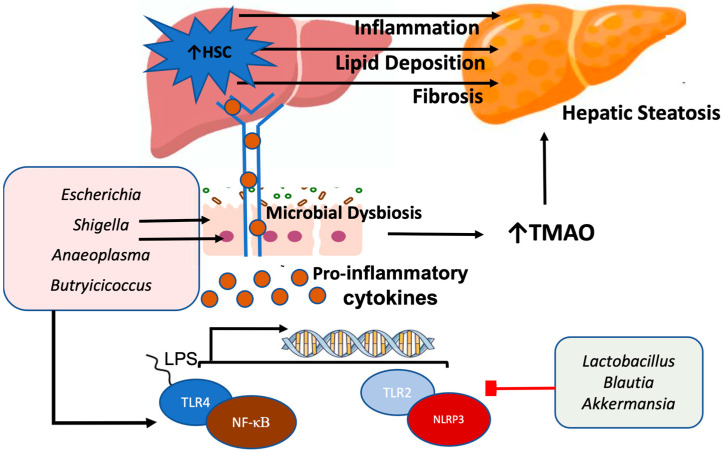
Gut microbiota and hepatic inflammation. Overabundance of enteric microbial species such as *Escherichia*, *Shigella*, *Anaeoplasma*, and *Butyricicoccus* up-regulate inflammatory pathways such as NF-κB and NLRP3. This occurs through LPS-mediated activation of TLR4 and lipoteichoic-acid-mediated activation of TLR-2. The resulting microbial dysbiosis concurrently leads to gut barrier permeability, increased unfavorable gut metabolites such as TMAO, and activation of pro-inflammatory cytokines that enter nearby systemic circulation via the portal venous system. Once in the liver, these pro-inflammatory cytokines may activate hepatic stellate cells, triggering hepatic inflammation, lipid deposition, and fibrosis. Over time, this drives steatotic changes within the liver. Conversely, increased abundances of favorable microbiota such as *Lactobacillus*, *Blautia*, and *Akkermansia* have been shown to mitigate these adverse effects. Abbreviations: HSC, hepatic stellate cells; TMAO, trimethylamine-N-oxide; LPS, lipopolysaccharides; TLR4, toll-like receptor 4; TL2, toll-like receptor 2; NLRP3, NOD-, LRR-, and pyrin-domain-containing protein 3.

Microbial dysbiosis contributes to liver inflammation and fat deposition through mechanisms that include increased gut barrier permeability; production of endotoxins; and activation of pro-inflammatory signaling pathways such as nuclear factor kappa beta (NF-κβ) and the nucleotide-binding domain, leucine-rich repeat, and pyrin-domain-containing protein 3 (NLRP3)-inflammasome [65]. Disruption of gut barrier integrity mediated by microbial interactions has been shown to exacerbate the severity of steatotic liver disease [66]. Specifically, distinct microbial signatures including alterations in *Escherichia*, *Shigella*, *Anaeoplasma*, and *Butyricicoccus* concentrations were associated with increased gut membrane permeability and steatosis through gut–liver interactions [66]. Gut microbial metabolites such as trimethylamine N-oxide (TMAO), which are elevated in individuals with steatotic liver disease, promote lipid deposition in hepatocytes by increasing intestinal barrier permeability and activating the toll-like receptor 4 (TLR4)/NF-κβ pathway [67]. Persistent activation of hepatocytic NF-κβ drives liver steatosis and supports de novo lipogenesis and cholesterol synthesis [68]. Conversely, beneficial gut microbial metabolites such as butyrate increase beneficial bacterial genera such as *Lactobacillus* and strengthen the intestinal barrier by up-regulating the expression of tight junction protein [69]. Enhanced gut barrier integrity reduces endotoxin leakage into the bloodstream, decreasing the amount of pro-inflammatory genes and cytokine-mediated inflammation from reaching the liver through the portal venous system (Figure 1) [38]. Amelioration of hepatic inflammation after strengthening of the gut barrier is also associated with decreased liver fat accumulation and lipid concentrations [69].

Further, increased abundances of Gram-negative bacteria, particularly those belonging to the *Enterobacteriaceae* family in the setting of dysbiosis, can contribute to metabolic endotoxemia and steatotic liver disease through the production of lipopolysaccharides (LPS) [70]. LPS bind to TLR4 on enterocytes, initiating signaling pathways that promote the release of pro-inflammatory cytokines and chemokines while increasing tight junction permeability, exacerbating intestinal barrier dysfunction [71]. In dysbiotic states with elevated levels of Proteobacteria, extracellular vesicles secreted by gut microbes exhibit increase expression of TLR4, macrophage markers, and cytokines that are transported via the hepatic portal vein to the liver amplifying inflammatory responses [72]. LPS-treated murine models showed significant lipid droplet accumulation in hepatocytes, accompanied by increased activity of enzymes involved in fatty acid synthesis, compared to controls [73]. Further, LPS challenge in hepatic steatosis models potentiated TLR4/NFκB signaling, leading to accelerated pericellular fibrosis in lipid-laden hepatocytes (Figure 1) [74]. Specifically, the activity of hepatic stellate cells (HSCs) is increased, amplifying hepatic inflammatory responses and promoting collagen-deposition in response to liver injury, contributing to fibrotic changes [75]. Chronic TLR4 activation exacerbates hepatic steatosis and fibrosis, and in advanced stages, may even contribute to hepatocarcinogenesis [76].

In addition to TLR4, toll-like receptor 2 (TLR2) is also implicated in steatohepatitis, particularly in the context of dysbiosis and Gram-positive bacterial cell wall components [77]. Overactivation of TLR2 stimulates Kupffer cells (liver macrophages) and HSCs, promoting the progression of steatotic liver disease. Kupffer cells in particular drive hepatic inflammation by activating the NLRP3 inflammasome [77]. Studies have shown that *Escherichia coli* strains isolated from the intestinal tract of steatotic liver disease patients can translocate to the liver via the TLR2/NLRP3 pathway. This translocation increases hepatic macrophage expression and exacerbates liver inflammation when introduced into the diet of murine models [78]. The NLRP3 inflammasome, an intracellular sensor responding to endogenous stimuli, such as gut-microbial-pathogen-associated molecular patterns (PAMPs), is a key driver of MASLD pathogenesis and progression when overactivated [79]. NLRP3 activation triggers cytokine release, increased reactive oxygen species formation, and lysosomal damage, facilitating inflammation in hepatocytes [80]. Further, NLRP3 activity induces hepatocytes to undergo pyroptosis, triggering cell death; release of inflammasome particles; and up-regulation of HSCs, which amplify liver fibrosis [81]. Given the correlation of NLRP3 with MASLD development, it has become a therapeutic target. For instance, supplementation with *Gynostemma pentaphyllum* polysaccharides has been shown to enrich beneficial bacteria such as *Akkermansia* and *Lactobacillus* while inhibiting TLR2 expression and down-regulating NLRP3 activity (Figure 1) [82]. Similarly, direct inhibition of NLRP3 has been shown to attenuate hepatic inflammation and fibrosis in murine models through suppressing cholesterol-mediated activation of Kupffer cells [83].

### 2.2. Gut Microbes, Hepatic Lipogenesis, and Steatotic Liver Disease

**Figure 2:**
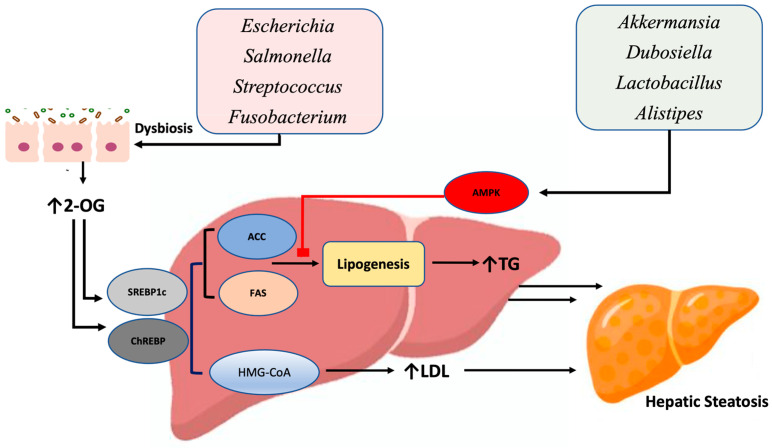
Gut microbial influence on lipogenesis and hepatic steatosis. Microbial dysbiosis induced by overgrowth of enteric bacterial genera including *Escherichia*, *Salmonella*, *Streptococcus*, and *Fusobacterium* leads to the overproduction of harmful bacterial metabolites such as 2-oleoylglycerol. These metabolites up-regulate lipogenic pathways in the liver. Specifically, expression of transcription factors SREBP1c and ChREBP are enhanced, which activates the lipogenic regulatory enzymes ACC and FAS. In turn, these enzymes increase triglyceride production and may result in fatty deposition in the liver. Alternatively, enhanced expression of SREBP1c and ChREBP increases cholesterol synthesis through increasing activity of HMG-CoA reductase, leading to increased formation of LDL, which may deposit in the liver to promote the development of hepatic steatosis. In contrast, favorable bacterial species such as *Akkermansia*, *Lactobacillus*, *Dubosiella*, and *Alistipes* may enhance AMPK expression, which inhibits lipogenic pathways to attenuate hepatic fat accumulation. Abbreviations: 2-OG, 2-oleoylglycerol; SREBP1c, sterol-regulatory-element-binding protein 1; ChREBP, carbohydrate-response-element-binding protein; ACC, acetyl-coenzyme A carboxylase; FAS, fatty acid synthase; HMG-CoA, 3-hydroxy-3-methylglutaryl coenzyme A; TG, triglycerides; LDL, low-density lipoprotein; AMPK, AMP-activated protein kinase.

Gut microbiota, in states of dysbiosis, are intricately linked to mechanisms that influence lipogenesis and fatty acid synthesis, contributing to hepatic fat deposition and progression of steatotic liver disease [84]. One key pathway involved is microbiota-mediated regulation of sterol-regulatory-element-binding proteins (SREBP1c), transcription factors that control lipid synthesis and adipogenesis [85]. SREBP1c control the primary enzymes that facilitate lipogenesis such as HMG-CoA synthase, fatty acid synthase (FAS), acetyl-CoA carboxylase (ACC), ATP citrate lyase (ACL), and stearoyl-CoA desaturase (SCD). For example, in murine models, the gut microbial metabolite 2-oleoyl glycerol (2-OG), which is elevated after high-fat-diet-induced obesity, has been shown to increase de novo lipogenesis through up-regulation of SREBP1c and carbohydrate-response-element-binding protein (ChREBP), another transcription factor that enhances lipogenic enzymes such as FAS and ACC (Figure 2) [86]. When 2-OG concentrations were measured in healthy subjects and those with MASLD, significantly higher levels were observed in MASLD patients, which may, in part, be due to excess lipid synthesis (Figure 2) [86]. Similarly, recent studies showed that diet-induced alterations in gut microbiota facilitate hepatic fat accumulation and lipogenesis through the SREBP1c/ACC/FAS pathway, an effect attenuated by the microbial transplant of beneficial microbial species [87].

In concert with these findings, AMP-activated protein kinase (AMPK), an inhibitor of lipogenesis that inactivates ACC through phosphorylation, is also modulated by microbial mechanisms to influence hepatic fat accumulation [88]. Six-week canola-oil-induced changes in gut microbiota enhanced AMPK phosphorylation activity, leading to suppressed lipogenesis in murine models [89]. Additionally, inhibition of SREBP1c as well as peroxisome-proliferator-activated receptor gamma (PPARγ) were observed after improvement of microbial diversity through increased abundances of *Akkermansia*, *Dubosiella*, and *Alistipes* (Figure 2). Notably, *Alistipes*-induced acetate production was correlated with AMPK phosphorylation, providing further insight into the interplay between microbiota and lipogenic pathways [89]. Further, beneficial bacterial strains such as *Lactobacillus* have been shown to activate AMPK signaling and reduce ACC expression, resulting in improved liver function, reduced circulating lipid concentrations, and inhibition of lipogenesis in a steatotic liver disease model [90]. These effects may be, in part, secondary to increased production of short-chain fatty acids (SCFA) by gut microbiota, as dietary-fiber-induced SCFA production activates AMPK-related genes involved in the gut–liver axis [91]. In addition to its negative regulation of fatty acid synthesis, AMPK also up-regulates β-oxidation to positively regulate utilization of fats in the liver and reduce lipid accumulation [91].

PPARγ is another regulator of lipogenesis that controls the gene expression of lipid transporters such as ATP-binding cassette transporter G1 (ABCG1). This transporter plays a critical role in cholesterol efflux from hepatocytes into the gallbladder, indirectly influencing bile acid homeostasis since cholesterol functions as a precursor for bile acid synthesis [92]. Though PPARγ is a known regulator of fatty acid redistribution, its role in steatotic liver disease is controversial. Some studies suggest that PPARγ overexpression promotes lipid efflux from hepatocytes to adipose tissues [93], while other studies showed a pro-steatotic role with PPARγ mediating increased fatty acid uptake in the liver [94]. However, PPARγ activity is significantly influenced by microbiota. For example, butyrate-induced expression of PPARγ prevents dysbiotic expansion of *Escherichia* and *Salmonella*, shifting the bioavailability of oxygen towards β-oxidation [95]. Further, tumor necrosis factor alpha (TNFα)- and interleukin-mediated inflammatory processes are shown to repress PPARγ activity, suggesting that low-grade inflammatory states such as those induced by microbial dysbiosis may influence PPARγ-mediated fatty acid redistribution [96]. Importantly, in steatotic liver disease, supplementation of beneficial *Lactoplantibacillus plantarum* improves PPARγ expression within its transcriptional network when introduced in high-fat-diet-induced MASLD murine models [97]. Taken together, these effects of PPARγ expression may be attributed to the robust butyrate-producing capabilities and anti-inflammatory effects of *Lactoplantibacillus*.

### 2.3. Gut Microbes, Bile Acid Metabolism, and Steatotic Liver Disease

Bile acids serve as essential regulators of the gut–liver axis, and their modification by gut microbiota have significant implications in steatotic liver disease [98]. Through enterohepatic circulation, bile acids are cycled between the liver and the terminal ileum, undergoing microbial-mediated modification within the intestinal tract in the process [99]. Functionally, bile acids are critical in fat digestion and absorption and serve as key modulators of lipid and glucose homeostasis [100]. In steatotic liver disease, both the concentration and subtypes of bile acids are profoundly altered within the enterohepatic circulation. These changes are associated with decreased signaling through bile acid receptors, such as Farsenoid X receptor (FXR) and Takeda G-protein receptor 5 (TGR5) [101]. FXR and TGR5 activation, with them being co-localized in enteroendocrine cells, initiates downstream signaling pathways to suppress MASLD pathogenesis by reducing liver fatty acid synthesis and uptake, resulting in overall improvements in glucose metabolism and cholesterol synthesis [98]. Notably, FXR and TGR5 agonists have been shown to reverse diet-induced hepatic steatosis, inflammation, and fibrosis, an effect mediated through up-regulation of mitochondrial function within hepatocytes [24].

**Figure 3:**
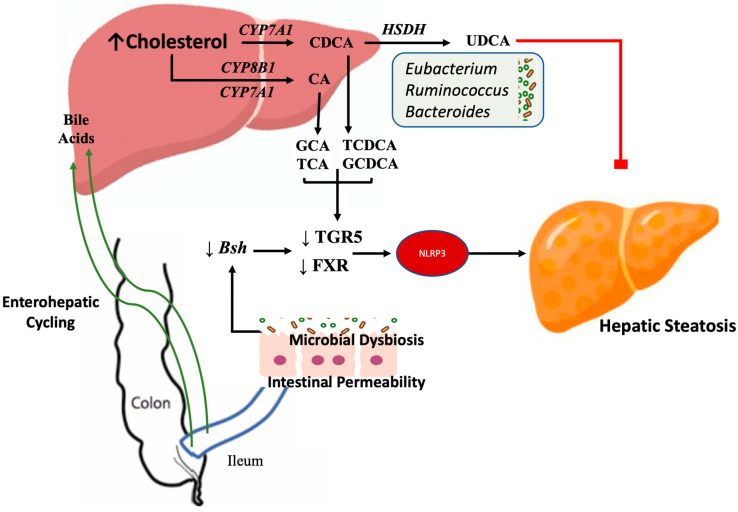
Gut microbiota influence on bile acid pool and impact on hepatic steatosis. Bile acids are formed from cholesterol derivates via enzymes such as CYP7A1 and CYP8B1. This enzymatic conversion to bile acids yields CDCA and CA, which may be further modified by sequential enzymatic reactions or within the intestinal tract by gut microbial species. Once formed, bile acids are secreted from the liver into the small intestine, traveling to the terminal ileum where around 95% are reabsorbed through the portal venous system and returned to the liver. Conversely, in the intestinal tract, bile acids are subject to conjugation, primarily with the addition of glycine to produce GCA and GDCA or taurine to produce TCA or TDCA prior to being returned to the liver. Increased conjugation of bile acids disrupts bile acid metabolism and worsens hepatic steatosis. In states of dysbiosis, microbes with bile salt hydrolase enzymatic activity are relatively reduced, diminishing bacterial ability to deconjugate these conjugated bile acids, further worsening the cycle. This also leads to decreased activation of TGR5 and FXR bile acid receptors, leading to the up-regulation of inflammatory signaling pathways and hepatic fat accumulation. Meanwhile, supplementation of beneficial bacteria such as *Eubacterium*, *Ruminococcus*, or *Bacteroides* is shown to promote UDCA formation from CDCA through inherent hydroxysteroid dehydrogenase activity. UDCA is beneficial in attenuating hepatic steatosis. Abbreviations: CYP7A1, cholesterol 7α-hydroxylase; CYP8B1, sterol 12-α-hydroxylase; CDCA, chenodeoxycholic acid; CA, cholic acid; HSDH, hydroxysteroid dehydrogenase; UDCA, ursodeoxycholic acid; GCA, glycocholic acid; TCA, taurocholic acid; TCDCA, taurochenodeoxycholic acid; GCDCA, glycochenodeoxycholic acid; TGR5, Takeda G protein-coupled receptor 5; FXR, Farsenoid X receptor; *Bsh*, bile salt hydrolase; NLRP3, NOD-, LRR-, and pyrin-domain-containing protein 3.

Recent findings have also shown that dysregulated bile acids contribute to the fibrotic progression of steatotic liver disease through NLRP3 inflammasome activation [102]. This mechanism is associated with decreased expression of FXR and accumulation of a toxic primary bile acid pool, leading to decreased phosphorylation and inactivation of the NLRP3 inflammasome [102]. Primary bile acids, significantly elevated in individuals with liver steatosis and fibrosis, include glycochenodeoxycholic acid (GCDCA), taurochenodeoxycholic acid (TCDCA), glycocholic acid (GCA), and taurocholic acid (TCA) [103]. In contrast, beneficial microbes such as *Akkermansia muciniphila* and *Bifidobacterium bifidum* prevent the onset of steatotic liver disease through regulation of FXR expression [104]. These microbial strains activated hepatic FXR while attenuating intestinal FXR signaling, resulting in reduced weight gain, improved insulin resistance, and decreased liver lipid deposition (Figure 3) [104]. Similarly, improvement of microbial composition through administration of dietary polysaccharides enhances bile acid pools by up-regulating production of secondary bile acids such as ursodeoxycholic acid (UDCA) and taurolithocholic acid (TLCA) [105]. These secondary bile acids have been associated with increased expression of FXR and TGR5, improving lipid metabolism and reducing steatotic fat deposition [105]. UDCA has a range of beneficial effects on liver fat accumulation through the activation of AMPK, mitigating oxidative stress and improving hepatic inflammation (Figure 3) [106].

Gut microbiota possess the enzymatic capability to influence bile acid metabolism through the activities of bile salt hydrolase (BSH) and hydroxysteroid dehydrogenases (HSDH), which carry out deconjugation and oxidation/epimerization reactions, respectively, on primary bile acids [107]. Reduced BSH activity negatively affects the bile acid pool, impairing FXR signaling and contributing to hepatic steatosis [108]. In contrast, BSH-overexpressing microbial species, such as *Lactobacillus casei*, attenuate hepatic steatosis, cholesterol accumulation, and lipid metabolism through reduced expression of SREBP1c, ACC, and FAS in steatotic liver disease murine models [109]. Similarly, HSDHs expressing bacterium such as *Eubacterium*, *Ruminococcus*, and *Bacteroides* genera contribute to the epimerization of bile acids to influence the total bile acid pool in MASLD (Figure 3) [110]. For example, biological synthesis of UDCA may occur through microbiota-mediated HSDH activity, which mediates oxidation, followed by epimerization reactions of chenodeoxycholic acid [111]. As previously mentioned, UDCA exerts multiple hepatoprotective effects and has demonstrated efficacy in humans as a preventative intervention for MASLD pathogenesis and progression [112]. Notably, besides via HSDH-mediated reactions, the body does not endogenously produce significant amounts of UDCA and therefore is often supplemented in certain liver conditions [111]. Collectively, these findings underscore the pivotal role of the gut–liver–bile axis, with gut microbiota serving as a key mediator of biochemical processes in hepatic steatosis.

### 2.4. Gut Microbes and Oxidative Stress in Hepatocytes

**Figure 4:**
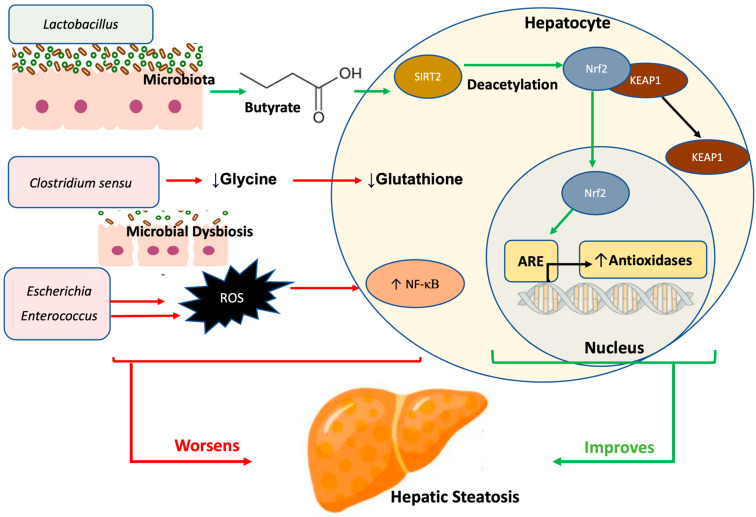
Oxidative stress, hepatocyte damage, and hepatic steatosis. *Lactobacillus* spp. improve microbial barrier through production of butyrate. Butyrate can increase sirtunin 2 activity, which influences nuclear-factor-erythroid-2-related factor 2 (Nrf2) through deacetylation reactions, allowing its dissociation from inhibitor Kelch-like ECH-associated protein 1. This allows Nrf2 to translocate into the nucleus, activate the antioxidase response element, and increase transcription of antioxidases to mitigate oxidative stress and thereby improve hepatic steatosis. Conversely, dysbiosis, observed through overabundance of *Clostridium sensu*, *Escherichia*, and *Enterococcus*, can deplete glycine, an important precursor of a main antioxidase, glutathione. Similarly, this can induce reactive oxygen species formation and the up-regulation of NF-κB-mediated inflammation. Therefore, dysbiosis can reduce antioxidase production, increase reactive oxygen species, and induce inflammation within hepatocytes to worsen hepatic steatosis. Abbreviations: SIRT2, sirtunin 2; Nrf2, nuclear-factor-erythroid-2-related factor 2; KEAP1, Kelch-like ECH-associated protein 1; ARE, antioxidase response element; ROS, reactive oxygen species; NF-κB, nuclear factor kappa beta.

The formation of reactive oxygen species (ROS) and related increases in oxidative stress are major contributors to liver fat accumulation through the induction of mitochondrial damage in hepatocytes [113]. ROS accumulation results from several biological processes associated with MASLD development including inflammatory signaling pathways, lipid peroxidation, and bile acid formation [114]. Therefore, dysregulation of these pathways through microbiota alterations plays a critical role in driving steatotic liver changes and hepatocyte damage [114]. For example, increased abundances of *Escherichia coli* and *Enterococcus* spp. have been positively correlated with ROS formation, while *Lactobacillus* spp. are negatively correlated with oxidative stress [115]. Although not specific to MASLD, *Lactobacillus* supplementation has been shown to up-regulate nuclear-factor-erythroid-2-related factor 2 (Nrf2), thus preventing hepatocyte injury caused by toxic metabolites such as ethanol and acetaminophen, which induce high levels of oxidative stress when ingested in excess (Figure 4) [116]. Nrf2 is an essential transcription factor that regulates synthesis of antioxidant enzymes and modulates cellular redox homeostasis. In steatotic liver disease, elevated NrF2 levels are observed as a compensatory response to increased oxidative stress [117].

In addition, Sirtunin 2 (SIRT2), a protein that can deacetylate Nrf2 by directly enhancing its nuclear localization, facilitates Nrf2 binding to antioxidant response elements and induces transcription of cytoprotective antioxidant genes (Figure 4) [118]. SIRT2 deficiency exacerbates diet-induced steatotic liver disease through direct influence on microbiota and their metabolites as evidenced by decreased *Bacteroides* and *Eubacterium*, with increased *Acetatifactor* [119]. These microbial shifts were associated with altered metabolites to promote lipid deposition and inflammation in hepatocytes, accelerating MASLD progression [119]. Conversely, interventions aimed at improving gut microbial profile such as herbal medications have been shown to enhance the abundance of SCFA-producing bacterial families such as *Christensenellaceae* and *Prevotellaceae* that positively regulate SIRT/Nrf2 signaling pathways in hepatocytes [120] leading to enhanced bile acid pool, increased SCFA concentrations, reduced oxidative stress, and decreased hepatic fat deposition [120]. These microbial metabolites are essential mediators of SIRT2 activity as SCFA, such as butyrate, influence its activity by increasing NAD+ concentrations, a cofactor for SIRT2 reactions, and through its inherent histone deacetylase inhibitor activity influencing epigenetic modulation of SIRT2 genes [121,122].

In murine models of MASLD, dysbiosis-associated species such as *Clostridium sensu* have been shown to alter bioavailability of glycine, a glutathione precursor, enhancing fatty liver deposition and fibrotic progression due to increased oxidative stress [123]. Supplementation of glycine in these dysbiotic mice attenuated NF-κβ-mediated hepatic inflammation, improved fatty acid oxidation, reduced lipotoxicity, and promoted glutathione synthesis (Figure 4) [123]. Glutathione, a potent antioxidant, plays an important role in mitigating cellular oxidative damage in hepatocytes, and its reduced activity has been implicated in the pathogenesis of liver disease [124]. Thus, the availability of glutathione precursors is essential for de novo glutathione synthesis and for preventing disease progression. Interestingly, interactions between Nrf2 and NF-κβ appear interrelated in modulating fatty liver development. For example, polysaccharide supplementation has been shown to influence expression of both transcription factors, effectively mitigating oxidative and inflammatory stress to alleviate hepatotoxicity and steatosis in murine models [125]. Taken together, these findings provide robust evidence linking gut microbiota metabolites, oxidative stress, and their impact on liver fat accumulation and MASLD progression.

## 3. Harmful Dietary Patterns for MASLD

**Figure 5:**
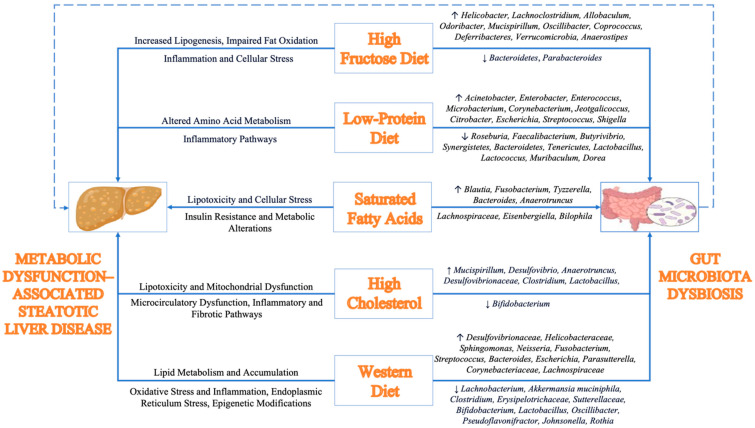
Effects of harmful dietary patterns on gut microbiota in MASLD. A high-fructose diet is shown to increase lipogenic pathways, impair fat oxidation, and induce inflammation and cellular stress. Low-protein diets deplete amino acid pools and induce inflammation. Saturated fatty acids confer lipotoxicity and cellular stress in hepatocytes while inducing insulin resistance. High-cholesterol diets also confer lipotoxicity, as well as mitochondrial and microcirculatory dysfunction. The Western diet impairs lipid metabolism, enhances lipid accumulation in the liver, and worsens oxidative stress and inflammation. Associated microbiota changes are shown within the figure. Abbreviations: ↑, increased abundance; ↓, decreased abundance.

Harmful dietary patterns negatively impact gut microbial composition, contributing to the development and progression of MASLD [126]. Diets characterized by high-caloric intake such as those rich in saturated fats, trans fats, cholesterol, and fructose-sweetened beverages, along with diets low in protein concentrations, promote lipid accumulation in the liver through microbiota-mediated mechanisms [127,128]. These changes serve as important factors in driving inflammation and progressive fibrosis [129]. Notably, the Western diet, which is marked by a high intake of processed foods, refined sugars, saturated fats, and animal proteins, coupled with a significant reduction in plant-based foods, have been shown to directly impacts liver health [130]. Processed foods, often containing additives and preservatives, disrupt gut microbiota balance, further exacerbating metabolic dysfunctions and promoting fat accumulation in the liver, ultimately progressing to non-alcoholic steatohepatitis [126,130]. In the following subsections, we explore the adverse effects of high-fructose, high-cholesterol, high-saturated-fat, and low-protein diets, focusing on their impact on gut microbiota and liver fat accumulation (Figure 5).

### 3.1. Highly Processed Fructose Diet, Gut Microbiota, and MASLD

Fructose, a naturally occurring monosaccharide, is commonly found in a variety of foods. Pome fruits, such as apples and pears, contain approximately 6 g of fructose per 100 g, while berries provide around 7.5 g per 100 g, and natural honey contains roughly 40 g per 100 g [131]. Fructose is also present in synthetic honey [132]. During food processing, fructose undergoes chemical reactions, such as polymerization and condensation, particularly when exposed to heat. These reactions generate compounds like aldehydes, reducing ketones, and heterocyclic compounds, contributing to food flavor through the Maillard reaction [133]. It is also important to differentiate between natural and processed sources of fructose [134]. Natural sources, as previously mentioned, are found in minimally processed fruits, honey, and other whole foods. These sources contain additional nutrients sources such as fiber that slows sugar absorption. Conversely, due to its intense sweetness, ability to enhance flavor, and high palatability, processed fructose is a frequent additive to processed foods and beverages. A leading example is high-fructose corn syrup (HFCS), widely used in soft drinks, preserved jams, breakfast cereals, and baked goods, playing a significant role in the profitability of the food industry [135,136,137]. Over the past four decades, fructose consumption has risen steadily among both adults and children. Globally, added sugars constitute approximately 15% of total daily energy intake with fructose, accounting for nearly half of this percentage [138]. Common sweeteners include sucrose, which contains 50% fructose, and HFCS, which can contain up to 55% fructose [139]. This increased incorporation of excessive fructose into diets has led to significant metabolic disadvantages with strong associations observed with steatotic liver disease, obesity, T2DM, and dyslipidemia [139]. While natural fructose from plant sources generally provides metabolic benefits largely due to the slower absorption and the presence of fiber and antioxidant, industrial fructose, such as HFCS and sucrose, particularly in liquid form, is rapidly absorbed and contributes to hepatic insulin resistance and MASLD. Notably, consumption of beverages with high HFCS content nearly triples the risk of developing MASLD [140].

Several experimental studies have highlighted the relationship between fructose intake and MASLD. In a study on female rats receiving isocaloric solutions of fructose or glucose for two months, only fructose led to hepatic steatosis without causing inflammation or oxidative stress. Additionally, fructose supplementation disrupted insulin signaling in major insulin-sensitive tissues, independent of increased caloric intake [141]. In another study, mice consuming fructose water (15% solution), sucrose (10%, a common soft drink), or artificial sweetener (0% calories) showed that fructose water significantly increased adiposity and induced lipid accumulation in the liver, despite no substantial change in total energy intake [142]. A study on Wistar rats compared the effects of fructose- and fat-enriched diets on non-alcoholic steatohepatitis. Rats fed a fructose-rich diet (70%) showed greater macrovesicular steatosis and lobular inflammation as well as higher hepatic triglyceride concentrations than rats maintained on high-fat (15%) or sucrose (70%) diets [143].

In clinical studies, a survey of 283 Lebanese adults reported an average daily fructose intake of approximately 51 g, with 12 g of fructose naturally found in foods and 39 g from added fructose [144]. This study also found an association between high total and added fructose consumption and increased risk of metabolic syndrome [143]. The metabolism of fructose exceeding 25 g/day is challenging for the body, particularly industrially processed fructose, which is rapidly absorbed and poses significant metabolic challenges [145]. Absorbed fructose stimulates hepatic lipogenesis, while unabsorbed fructose disrupts metabolic balance, contributing to MASLD progression [146]. Ouyang et al. [146] found that patients with steatotic liver disease consumed 2–3 times more fructose than control subjects, suggesting that excessive fructose consumption plays a significant role in MASLD development. In another study, 80% of steatotic liver disease patients consumed more than 500 mL/day of soft drinks, compared to just 17% of healthy individuals. These patients derived 40% of their carbohydrates from soft drinks, compared to only 8% in healthy individuals [147]. The study also found that high consumption of these beverages was an independent predictor of fatty liver, regardless of metabolic syndrome status [147]. Conversely, a pilot study showed that reduction of fructose intake by 50% in patients with steatotic liver disease showed significantly decreased hepatic lipid content, plasma transaminase levels, Body Mass Index, fasting plasma insulin, endotoxin concentrations, and plasma plasminogen activator inhibitor-1 (PAI-1) after six months [148].

Several mechanisms have been proposed to explain how a high-fructose diet promotes MASLD. Fructose consumption drives hepatic fat accumulation by serving as both a substrate and an inducer of de novo hepatic lipogenesis, leading to increased fat production in the liver [149]. This process is facilitated by the activation of key transcription factors such as ChREBP and SREBP1c, which regulate genes involved in gluconeogenesis and lipogenesis, both of which are activated by fructose metabolism [150]. Another proposed mechanism involves the disruption of fatty acid oxidation. Fructose metabolism via fructokinase C consumes ATP, reducing the energy available for lipid oxidation, thus promoting fat accumulation in the liver [151]. Additionally, fructose increases malonyl-CoA levels, which inhibits fatty acid oxidation and further amplifies fat deposition, contributing to hepatic steatosis [152]. Fructose also triggers hepatic inflammation, cellular stress, oxidative stress, and endoplasmic reticulum stress, all of which accelerate disease progression [149]. Notably, Li et al. identified Usp2 as a fructose-sensitive gene in the liver, with elevated levels of USP2 detected in hepatocytes of mice with MASLD, as well as in primary hepatocytes exposed to fructose [140]. USP2 is a regulator of hepatic gluconeogenesis, and therefore overexpression of USP2 amplified lipid accumulation, glucose intolerance, and metabolic inflammation, while reducing its expression mitigated fructose-induced effects [140,153].

A study on mice with steatotic liver disease showed that a long-term diet high in fats and fructose increased the abundance of bacterial genera such as *Helicobacter*, *Faecalibaculum*, *Lachnoclostridium*, *Allobaculum*, *Odoribacter*, and *Mucispirillum* [154]. These bacteria were associated with MASLD pathogenesis, obesity, oxidative stress, and intestinal inflammation, with some, like *Allobaculum* and *Odoribacter*, potentially increasing the risk of diabetes, exacerbating hepatic fat accumulation [154]. Another study on mice fed a fructose-rich diet for 12 weeks reported a decreased Bacteroidetes/Firmicutes ratio and an increase in *Blautia*, *Lachnoclostridium*, and *Oscillibacter* [155]. Several studies have shown that fructose intake increased the abundance of bacterial strains like *Coprococcus* and *Ruminococcus* [156], raising the Firmicutes/Bacteroidetes ratio [157,158,159], and reduced the abundance of the *Bacteroidetes* phylum [157]. Additionally, fructose consumption has been linked to increased levels of pathogenic bacteria, including *Deferribacteraceae* and *Helicobacteraceae* [159]; a decrease in Actinobacteria diversity in rats [158]; and an increase in *Deferribacteres*, *Verrucomicrobia*, and *Actinobacteria* phyla along with higher levels of *Bacteroides*, *Akkermansia*, and *Ruminococcus* genera [159].

In a study involving 12 healthy women, a high-fructose diet (100 g/day) led to an increase in Firmicutes and beneficial butyrate-producing bacteria such as *Faecalibacterium*, *Anaerostipes*, and *Erysipelatoclostridium*, while it decreased Bacteroidetes and *Parabacteroides* [160]. However, a diet supplemented with fructose syrup (100 g/day) resulted in a decrease in Firmicutes and *Ruminococcus*, while increasing Bacteroidetes, compared to a fruit-rich diet. Compared to a low-fructose diet (<10 g/day), the fructose-syrup-supplemented diet led to a decrease in *Faecalibacterium* and *Erysipelatoclostridium* [160]. These findings collectively suggest that excessive fructose consumption, particularly from industrial sources, induces an imbalance in the gut microbiota, reducing beneficial commensal microorganisms that help maintain intestinal barrier integrity. These changes contribute to increased intestinal permeability, leading to endotoxemia and excessive nutrient absorption, which in turn promotes hepatic inflammation and fat accumulation in the liver [146]. In summary, the cinsumption of processed sources of fructose has been shown to exert detrimental effects on hepatic pathophysiology. Therefore limiting the intake of HFCS, industrial sweeteners, and sucrose is an important strategy for attenuating the progression of hepatic steatosis.

### 3.2. Low-Protein Diet, Gut Microbiota, and MASLD

Research has shown significant associations between protein-deficient, carbohydrate-rich diets, which are commonly seen in vulnerable populations, and hepatic lipid accumulation. For example, severely malnourished children not only exhibit hepatic steatosis but also experience other metabolic disturbances and increased oxidative stress [161]. The development and maintenance of muscle mass are closely dependent on adequate protein intake. While the recommended daily intake for healthy adults is 0.8 g/kg body weight, individuals with sarcopenia, the elderly, and patients with various conditions such as liver or lung diseases require higher protein intakes to preserve muscle mass. It is recommended that these individuals increase their protein intake to 1.2–1.5 g/kg body weight [162].

In animal studies, rats on a low-protein diet (8%) for four weeks developed hepatic steatosis, which was associated with increased enzyme activity involved in fat synthesis and a reduction in lipoprotein secretion, responsible for transporting fats from the liver [163]. Another study on weaned rats fed different diets for 30 days showed that a low-protein diet (3%) led to hepatic steatosis. Interestingly, supplementing this diet with medium-chain triglycerides resulted in a reduction in hepatic fat deposits [164]. A low-protein diet inhibits the activation of the PPARα gene, which plays a critical role in regulating hepatic metabolism during nutritional restriction. Laboratory research has shown that fish protein supplementation restores expression of PPARα target genes, such as *ACOX1* and *CPT2*, stimulating fatty acid oxidation in the liver and providing hepatoprotection [165]. Protein deficiency can also impair liver function by disrupting the amino acid profile, particularly by reducing glycine and serine levels, which are essential for liver function and metabolic processes [166]. Additionally, insufficient protein intake alters plasma concentrations of amino acids, such as branched chain amino acids, which are linked to increased fat accumulation in the liver [167,168]. Furthermore, low-protein diets can affect lipolytic and lipogenic pathways in the liver and adipose tissue, promoting liver fat accumulation and inflammation [169]. These diets also influence inflammatory markers such as CRP, TNF-α, and IL-6, which are implicated in the development and progression of steatotic liver disease, contributing to hepatic inflammation and fibrosis [169,170].

A study by Masuoka et al. on pathogen-free mice fed diets with varying protein content (3%, 6%, 9%, or 12%) for four weeks showed that mice on the 3% protein diet had increased abundance of *Actinobacteria*, *Proteobacteria*, *Acinetobacter*, *Enterobacter*, *Enterococcus*, *Microbacterium*, *Corynebacterium*, *Jeotgalicoccus*, and *Citrobacter*, while the abundance of *Bacteroidetes*, *Tenericutes*, *Lactobacillus*, *Lactococcus*, *Muribaculum*, and *Dorea* decreased [171]. In a different clinical study conducted on 20 children, with varying nutritional statuses, better nutritional status was positively correlated with increased abundance of *Roseburia*, *Faecalibacterium*, *Butyrivibrio*, and the phylum Synergistetes. Conversely, poorer nutritional status was associated with a higher abundance of potentially pathogenic bacteria including *Escherichia*, *Streptococcus*, *Shigella*, *Enterobacter*, *Veillonella*, and the phylum Proteobacteria [161]. Notably, protein supplementation in steatotic liver disease models was associated with improvements in gut microbial composition, reversing dysbiosis associated with nutritional deficiencies [172].

In summary, states of protein deficiency shift metabolic focus to amino acids as a source of energy, leading to liver fat accumulation and inflammation. These inflammatory changes are partly attributed to microbiota imbalance during nutritional deficiency as reduced availability of substrates for beneficial bacteria leads to the overproduction of harmful metabolites [173]. Consequently, individuals with MASLD require higher protein intake (1.2–1.5 g of protein per kilogram of body weight) than those without liver disease, given the inherent propensity of muscle wasting associated with liver dysfunction [174]. Therefore, monitoring protein intake is an important component of preventing liver disease and is recommended in individuals with ongoing liver dysfunction to support hepatic function.

### 3.3. Saturated Fatty Acids, Gut Microbiota, and MASLD

Foods rich in saturated fats include both animal-based products, such as cream, butter, whole dairy, and fatty meats, as well as some plant-based oils like coconut oil and palm kernel oil. Additionally, many processed foods, such as pizza, dairy desserts, and sausages, are significant sources of saturated fats [175]. Excessive consumption of these fats is linked to insulin resistance and elevated LDL cholesterol levels, both of which negatively impact metabolic health [176].

A cross-sectional nationwide study using data from the 2017–2018 National Health and Nutrition Examination Survey (NHANES) found that daily intake of saturated fatty acids was 29.7 g in patients with MASLD without advanced fibrosis and 34 g in those with advanced fibrosis compared to 27.9 g in the control group [177]. Similarly, a study involving 160 patients with steatotic liver disease and 160 healthy controls found that steatotic liver disease patients consumed substantially more saturated fatty acids than the control group. Their intake correlated with an increased risk of steatotic liver disease, especially when it exceeded 8% of total daily calories. High saturated fat intake was also associated with increased hepatic fat content, elevated liver enzymes, higher atherogenic lipids levels, and elevated plasma ceramides [178]. Further, Luukkonen et al. demonstrated that saturated fat supplementation in overweight individuals led to the greatest increase in intrahepatic triglycerides, promoting insulin resistance and the harmful accumulation of ceramides [179]. Similarly, a study on overweight and obese patients confirmed these findings, showing that saturated fat supplementation significantly increased hepatic fat content and elevated liver enzymes, atherogenic lipids, and circulating ceramides [180]. Experimental studies have shown that the combination of saturated fatty acids and dietary cholesterol exacerbates hepatic inflammation and metabolic dysfunction, accelerating MASLD progression and fibrotic changes [181]. Consequently, reducing saturated fat and cholesterol intake may represent an effective therapeutic strategy for MASLD, potentially improving lipid profiles and reducing cardiovascular risk [182].

The link between saturated fatty acid intake and MASLD development involves several mechanisms, including lipotoxicity and cellular stress. Elevated levels of saturated fatty acids can cause endoplasmic reticulum stress, triggering pathways that lead to hepatocyte death and impaired liver function, key contributors to the development of steatotic liver disease [183]. Additionally, saturated fatty acids impact mitochondrial efficiency, promoting the formation of reactive oxygen species, which damage cellular structures and promote inflammation, cell death, and liver fibrosis [183]. Saturated fatty acids can also interact with lipopolysaccharides, amplifying inflammatory responses in hepatocytes and increasing the production of pro-inflammatory cytokines, such as IL-6, to accelerate disease progression [184]. These fats disrupt energy homeostasis by altering carbohydrate and lipid metabolism, exacerbating metabolic syndrome and predisposing individuals to MASLD [182]. Further, excessive intake of saturated fatty acids rapidly induces insulin resistance in the liver and adipose tissue, disrupting energy balance and promoting hepatic fat accumulation. This metabolic shift is mediated by epigenetic mechanisms that up-regulate genes involved in hepatic lipogenesis such as PPARγ, very low-density lipoprotein receptor (VLDLR), and CD36, contributing to the onset of steatotic liver disease and intensifying the effects of metabolic syndrome [185,186].

High saturated fat intake has been associated with an increased Firmicutes/Bacteroidetes ratio, a marker linked to several pathological conditions, including obesity, T2DM, and other metabolic diseases [187]. In a study of 531 Finnish men, the abundance of the *Blautia* genus was positively correlated with the consumption of both saturated and monounsaturated fatty acids [188]. Similarly, in overweight and obese women, saturated fat intake was negatively associated with gut microbiota diversity and richness [189]. Other studies reported associations between saturated fat intake and increased abundance of genera such as *Fusobacterium* and *Tyzzerella* in individuals with normal colon findings [190], and with *Anaerotruncus*, *Lachnospiraceae*, *Eisenbergiella*, *Flavonifractor*, *Campylobacter*, and *Erysipelotrichaceae* in healthy subjects [191]. In experimental models, mice fed a lard-based diet for 11 weeks had increased abundance of *Bacteroides*, *Turicibacter*, and *Bilophila* genera [192]. These microbial alterations, driven by high saturated fat intake, may contribute to systemic inflammation, metabolic dysfunction, and the progression of MASLD. Therefore, limiting intake of saturated fats is an important factor in microbial and liver health.

### 3.4. High Cholesterol, Gut Microbiota, and MASLD

Major dietary sources of cholesterol include egg yolks, shrimp, beef, pork, and poultry, as well as cheese and butter [193]. Recent research suggests that excessive cholesterol intake may significantly contribute to the growing prevalence of MASLD [193]. For example, in a study of 1648 MASLD patients and 2527 control individuals, daily cholesterol intake for those with MASLD without advanced fibrosis was 305.4 mg, while those with advanced fibrosis consumed 413.9 mg compared to 293.6 mg intake in the control group [177]. Likewise, Musso et al. reported that patients with steatotic liver disease consumed 506 mg of cholesterol per day, significantly more than the 405 mg/day consumed by controls [194].

In a 2015 case–control study, consuming 2–3 eggs per week was associated with a 3.56-fold increased risk of developing steatotic liver disease compared to consuming fewer than two eggs per week. After adjusting for known risk factors, this risk increased to 3.71-fold [195]. Similarly, a large cohort study involving 14,369 participants found that individuals with the highest egg consumption had an 11% higher risk of hepatic fat accumulation compared to those with the lowest consumption [196]. The Golestan Cohort Study also identified a positive association between red and organ meat consumption and steatotic liver disease risk [197]. Furthermore, a case–control analysis linked steatotic liver disease development to high cholesterol intake and the consumption of red meat, processed red meat, and poultry [198].

Several experimental studies have explored the effects of cholesterol consumption on MASLD. Mells et al. showed that increased dietary cholesterol intake significantly elevated serum leptin and IL-6, liver weight, and fibrosis, as well as up-regulating smooth muscle α-actin in adult male mice [199]. In another study, mice fed a high-fat diet containing 1.25% cholesterol and 0.5% cholic acid, combined with 2% hydroxypropyl-β-cyclodextrin in drinking water, developed steatotic liver changes and insulin resistance within three weeks [200,201]. Research conducted over 14 months examining the effects of various diets in male mice found that high cholesterol intake promoted the progression from steatosis to steatohepatitis, fibrosis, and eventually hepatocellular carcinoma. These effects were associated with insulin resistance and gut microbiota changes, including increased abundance of *Mucispirillum*, *Desulfovibrio*, *Anaerotruncus*, and *Desulfovibrionaceae* families along with reduced populations of *Bifidobacterium* and *Bacteroides*. Germ-free mice transplanted with microbiota from cholesterol-fed mice also developed hepatic lipid accumulation, inflammation, and cellular proliferation [202]. A 2023 study further demonstrated the role of cholesterol in MASLD, demonstrating increased BSH-activity in bacteria such as *Bacteroides*, *Clostridium*, and *Lactobacillus* in mice fed a high-fat, high-cholesterol diet [203]. A randomized controlled trial involving 49 healthy participants also found a positive correlation between cholesterol intake and the abundance of *Proteobacteria*, a bacterial group linked to low-grade inflammation [204].

Excess accumulation of cholesterol in the liver plays a critical role in MASLD progression. The resultant lipotoxicity, a toxic chain reaction, compromises mitochondrial function and reduces liver cell regenerative capacity. The process involves formation of oxysterols, which further amplifies mitochondrial damage, impairs cellular energy production, and promotes the transition from hepatic steatosis to inflammation [205]. In addition, cholesterol disrupts cellular functions, induces inflammation, and impairs microscopic blood circulation in the liver. This process involves mitochondrial and lysosomal dysfunction; the formation of cholesterol crystals that damage hepatocytes; and activation of the transcription factor HIF-1α, which worsens inflammation and hepatic fibrosis. This cascade of events reduces liver blood flow, further aggravating liver damage [206,207]. These findings underscore the detrimental effects of excessive dietary cholesterol on liver health and its significant role in MASLD pathogenesis, emphasizing the importance of dietary modification as a preventive and therapeutic strategy.

### 3.5. Western Diet, Gut Microbiota, and MASLD

The Western diet is characterized by excessive consumption of processed and refined foods, including fast food, sugary carbonated drinks, and packaged snacks. These foods are typically high in added sugars, salt, unhealthy fats, and additives designed to enhance flavor and extend shelf life. In addition, the diet includes a high intake of red and processed meats, as well as saturated and trans fats [208]. A case–control study involving 320 participants found that adherence to the Western dietary pattern was a significant risk factor for steatotic liver disease [209]. In addition, a recent 2024 study comparing the effects of a low-carbohydrate, high-fat diet with a Western diet rich in fats and sugars in male C57BL/6J mice further confirmed that the Western diet led to obesity, glucose intolerance, elevated insulin levels, and the onset of MASLD [210]. Mirmiran et al. observed a positive association between high adherence to the Western diet and increased risk of hepatic fat accumulation with participants exhibiting a 2.6-fold greater likelihood of developing steatotic liver disease compared to those with low adherence [211]. Additional studies further corroborate the relationship between the Western diet pattern and elevated risk of steatotic liver disease [212,213].

Further, a six-month study on male C57BL/6 mice reported extensive macro- and microvesicular hepatic steatosis; pericellular fibrosis; hepatic stellate cell activation; infiltration of CD68+ macrophages; and increased pro-inflammatory proteins such NF-κβ (p65), IL-6, and TNF-α. This was accompanied by a reduction in the antioxidant regulator Nrf2 [214]. The study also showed decreased bacterial diversity, increased abundance of *Firmicutes* and *Proteobacteria*, a reduction in *Bacteroidetes* and *Fusobacteria*, and an altered Firmicutes/Bacteroidetes ratio [214]. Further studies have corroborated these findings, showing that the Western diet exacerbates liver inflammation through the activation of pro-inflammatory molecules such as IL-6 and TNF-α while reducing the liver’s antioxidant capacity [215,216,217]. These pro-inflammatory effects induce endoplasmic reticulum stress, further aggravating cellular damage and promoting hepatic fibrosis [217]. Notably, supplementation with amino acids in murine models adhering to a Western diet was shown to decrease lipid deposition, reduce hepatic inflammation, preserve antioxidant status, and increase the levels of *Bacteroides/Prevotella*. This suggests that some protective effects of amino acid supplementation may be mediated through gut microbiota [217].

High added sugar consumption associated with the Western diet increases Firmicutes/Bacteroidetes ratio and reduces butyrate-producing bacteria, such as *Lachnobacterium* [218]. In contrast, consumption of refined palm oil appears to negatively affect gut microbiota by reducing beneficial bacteria like *Akkermansia muciniphila*, SFB, and *Clostridium leptum* [219]. On the other hand, refined olive oil (as opposed to extra virgin olive oil) has been associated with an increase in the abundance of bacterial families such as *Desulfovibrionaceae*, *Spiroplasmataceae*, and *Helicobacteraceae*, while decreasing the abundance of *Erysipelotrichaceae* and *Sutterellaceae* [220]. Sunflower oil increases the abundance of *Sphingomonas* and *Neisseria* spp. while reducing beneficial bacteria such as *Akkermansia muciniphila* and *Bifidobacterium* [221]. High red meat consumption has been linked to an increased abundance of *Fusobacterium nucleatum*, *Streptococcus bovis/gallolyticus*, *Escherichia coli*, and *Bacteroides fragilis* [222]. High salt intake has been associated with a decrease in the abundance of *Lactobacillus* sp., *Oscillibacter*, *Pseudoflavonifractor*, *Clostridium XIVa*, *Johnsonella*, and *Rothia* while increasing the abundance of *Parasutterella* spp., the *Erwinia* genus, *Christensenellaceae*, *Corynebacteriaceae*, *Lachnospiraceae*, and *Ruminococcus* [223,224,225].

Collectively, these changes are indicative of gut dysbiosis, increased intestinal permeability, and bacterial translocation, leading to elevated endotoxins reaching the liver, causing inflammation and fibrosis and the progression of MASLD, highlighting the influence of the Western diet on disease pathogenesis. Moreover, the Western diet combines several harmful food components including fructose, saturated fats, and high cholesterol, which collectively exert compounding effects on gut microbiota and liver fat accumulation. Therefore, avoiding food sources characteristic of the Western diet and adhering to the healthier dietary patterns outlined in Section 4 is an important recommendation for preserving liver health.

## 4. Healthy Dietary Patterns and MASLD

**Figure 6:**
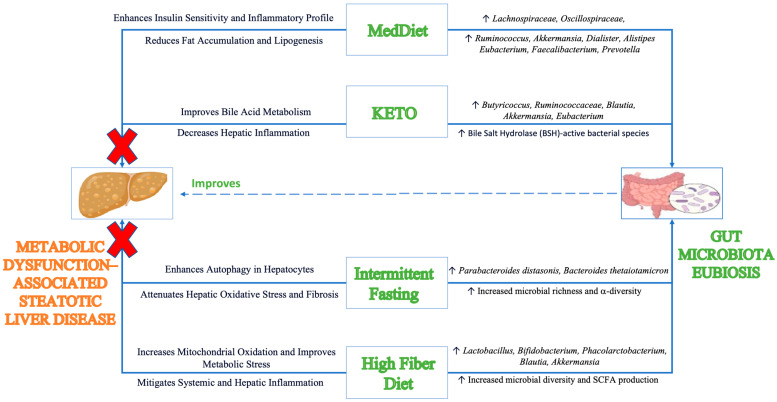
Effects of beneficial dietary patterns on gut microbiota in MASLD. The Mediterranean diet is shown to improve systemic inflammation and improve metabolic parameters, observed through increased insulin sensitivity with the reduction of lipogenesis and associated hepatic fat accumulation. A ketogenic diet is shown to increase abundances of BSH-active bacterial species, thereby improving bile acid metabolism, improving MASLD parameters. Intermittent fasting enhances autophagy; clears excess lipid droplets from hepatocytes; and reduces oxidative stress, inflammation, and fibrosis. Diets high in fiber augment mitochondrial function and attenuate metabolic stress while mitigating systemic and hepatic inflammation. The microbial changes from these dietary interventions can slow the progression of MASLD through these mechanisms. Abbreviations: MedDiet, Mediterranean diet; KETO, ketogenic diet; ↑, increased abundance; ↓, decreased abundance, X, mitigates or reduces.

Healthy dietary patterns have been studied for their role in improving metabolic parameters with modulation of gut microbiota and their metabolites as key factors in driving their beneficial effects [226] (Table 1). Among these are the low-glycemic-index Mediterranean diet, the ketogenic diet, intermittent fasting, and a high-fiber diet [227]. In the following subsections, we explore the general composition of each of these diets, their effects on gut microbiota, and how these microbial changes contribute to reducing liver fat accumulation and mitigating the progression of steatotic liver disease (Figure 6).

### 4.1. Mediterranean Diet, Gut Microbiota, and MASLD

The Mediterranean diet (MedDiet) is a low-glycemic, plant-based diet rich in nuts, whole grains, wheats, fruits, vegetables, olive oils, fibers, and polyphenols [250]. This nutrient-dense diet has demonstrated substantial benefits in managing metabolic disease, including MASLD, through the reduction of hepatic fat accumulation, improvement of insulin sensitivity, promotion of anti-inflammatory effects, and mitigation of progression of liver fibrosis [12,230]. In general, hepatic steatosis is shown to be inversely correlated with adherence to the MedDiet [251]. For example, a recent retrospective study of 5395 individuals showed that higher adherence to the MedDiet was associated with lower rates of steatotic liver disease, along with reduced triglyceride levels and improved glucose indices [228]. A meta-analysis of 10 randomized controlled trials involving 737 adult patients with steatotic liver disease showed that the MedDiet adherence resulted in reduced liver stiffness and total cholesterol levels, with intervention durations ranging from 6 weeks to 1 year [229]. Another meta-analysis further demonstrated dose-dependent improvements in liver enzymes, fatty liver index, and fibrosis with increased caloric restriction following the MedDiet [230]. Similarly, the MedDiet enhances fatty acid profiles, reversing the effects of high-fat dietary habits, decreasing proinflammatory markers, and improving oxidative stress in patients with steatotic liver disease [12]. These effects of the MedDiet are augmented when combined with regular physical activity [252].

The MedDiet’s ability to modulate gut microbial composition plays a crucial role in mediating its beneficial effects on steatotic liver disease [231]. Stool sample analysis of patients with steatotic liver disease who adhered to the MedDiet for 90 days showed increased abundances of *Rumminococcacae*, *Lachnospiraceae*, and *Oscillospiraceae*, as well as the genera *Ruminococcus*, *Akkermansia*, *Dialister*, *Alistipes*, and *Eubacterium* [231]. These changes contribute to improved outcome in hepatic steatosis. For example, the *Rumminococcacae* family is inversely associated with fibrosis severity in non-obese individuals with steatotic liver disease, and its sub-genera *Ruminococcus* reduces liver damage through decreases in liver enzymes [253]. The beneficial effects of *Rumminococcacae* are associated with the bile salt hydrolase (*bsh*) gene, an important mediator of bile salt deconjugation and maintenance of beneficial bile acid pools. Increased *bsh* expression improves hepatic steatosis through multiple mechanisms including enhanced FXR signaling, cholesterol homeostasis, and inhibition of liver fat deposition [109,254]. Along with *Rumminococcacae*, elevation of *Akkermansia* symbiotically enhances gut barrier integrity through the increased expression of tight junction proteins and reinforcing thickness of the mucous lining of the gut epithelium to mitigate metabolic endotoxemia [255]. Further, *Alistipes*, inversely associated with MASLD pathogenesis parameters [40], may mediate beneficial effects of the MedDiet through the production of acetate and propionate to mitigate hepatic inflammation [256]. The PREDIMED-Plus trial further supports these findings, showing that one year of MedDiet adherence improved microbial diversity and positively influenced Fibrosis-4 (FIB-4) scores, with the most significant effects observed in individuals with early-stage fibrosis [232].

A systematic review of 37 studies demonstrated consistent increases in *Faecalibacterium* and *Prevotella* with MedDiet adherence, leading to improved glycemic control, reduced steatosis, and decreased low-grade systemic inflammation [233]. Murine models of steatotic liver disease have shown that *Faecalibacterium* strains mitigate disease, improving glucose tolerance, adipose tissue function, and liver fat accumulation [257]. Structural abnormalities of hepatic lobules, indicated by intracytoplasmic lipid, were improved following *Faecalibacterium* introduction [257]. Additionally, lower mRNA expression of proinflammatory cytokines including TNF-α and IL-6 along with increased activity of superoxide dismutase and glutathione peroxidase were observed in *Faecalibacterium*-treated groups, indicating an improved inflammatory and oxidative profile [257]. These beneficial effects can be attributed, in part, to the SCFA-producing capability of *Faecalibacterium*, resulting in elevated concentrations of acetate, propionate, and butyrate, which are crucial in maintaining hepatic energy homeostasis and lipid metabolism [258]. Another study further elucidated the mechanisms by which increased *Faecalibacterium* alleviates MASLD [259]. In rodent models, human-derived *Faecalibacterium* strains prevented liver fibrosis by reducing lobular inflammation, up-regulating tight junction proteins such as zonulin-1 and occludin to strengthen intestinal epithelial barrier integrity and attenuating LPS/TLR4 expression to decrease metabolic endotoxemia [259]. Elevations in *Prevotella* following MedDiet adherence may also reduce hepatic fat accumulation. Individuals with Prevotella-dominant enterotypes are more responsive to dietary interventions, losing nearly double the weight compared to those with Bacteroides-dominant enterotypes [47]. This weight loss effect, coupled with the metabolic benefits of *Faecalibacterium*, underscores the potential of gut microbiota modulation in improving metabolic parameters and slowing the progression of MASLD through adherence to the MedDiet.

### 4.2. Ketogenic Diet, Gut Microbiota, and MASLD

The ketogenic diet (KETO) emphasizes low carbohydrate (5–10% of total caloric intake), high fat (55–60% of total caloric intake), and moderate-to-high protein consumption (30–35% of total caloric intake) [260]. Energy sources such as sucrose, glucose, and starches are avoided, which shifts the body’s primary energy source from glucose to fat, inducing a metabolic state known as ketosis [261]. This state promotes fat utilization, confers anti-inflammatory effects, improves insulin sensitivity, and reduces liver fat accumulation, thus improving outcomes in patients with MASLD [262]. Ketone body metabolism, a hallmark of steatotic liver disease and compensatory mechanisms in early stages of liver fat accumulation, alleviates metabolic stress by converting excess fatty acids into ketone bodies such as β-hydroxybutyrate and acetoacetate [263]. Ketogenesis occurs in the mitochondria of liver cells and involves a series of enzymatic reactions that break down fatty acids through β-oxidation. With the development of fibrotic damage to hepatocytes due to disease progression, ketogenesis is dampened and ketone bodies are reduced [264]. This is, in part, due to elevated insulin levels and associated insulin resistance, which is a potent inhibitor of ketone body formation. By promoting glucose uptake into cells, excess insulin mitigates fat breakdown and inhibits the activity of HMG-CoA synthase, the rate-limiting step of ketogenesis, leading to a cycle of fat deposition within hepatocytes [265]. Interestingly, a recent study has shown that acute insulin secretion following a ketogenic diet meal is 17 times lower compared to a meal following a MedDiet [266]. Therefore, KETO, when managed carefully, contributes to fat breakdown and enhanced insulin signaling, thus counteracting the onset and progression of steatotic liver disease [267] while concomitantly improving associated comorbidities like obesity and T2DM.

For example, adherence to a KETO for 6 days in patients with steatotic liver disease decreased intrahepatic triglycerides by 31% and hepatic insulin resistance by 58% as measured via isotopomer nuclear magnetic resonance tracing [234]. In this short study trial, there was a 238% increase in the partitioning of fatty acids towards ketogenesis, which was directly correlated with a ~50% drop in insulin levels and hepatic citrate synthase flux. This also resulted in a 167% improvement in the hepatic mitochondrial redox state, allowing for fatty acid breakdown through the optimization of the hepatic microenvironment for β-oxidation [234]. In murine models, an eight-week KETO regimen suppressed inflammatory markers (e.g., TNF-α, NF-κβ, IL-6, and TLR4) and enhanced mitochondrial function, reducing oxidative stress and inflammation through inhibiting the TLR4/NLRP3 inflammasome pathway [268,269]. A prospective study involving 87 overweight and obese patients with MASLD revealed significant improvements after an eight-week KETO intervention, including reduced white blood cell count, total cholesterol, insulin resistance, and liver fibrosis, as measured by FibroScan [235]. Interestingly, there were differences in gender specific responses, with men exhibited higher baseline liver steatosis and showing greater reductions in CRP and liver fibrosis post-intervention, while other metabolic parameters improved similarly across genders [270]. Additionally, individuals with more advanced steatosis experienced greater improvements following KETO adherence [271].

The ketogenic diet positively influences gut microbiota composition, increasing beneficial genera such as *Akkermansia* and *Eubacterium* [272]. Additionally, other SCFA-producing genera including *Butyricicoccus* and *Blautia*, along with elevated *Rumminococcacae*, have been observed following low-carbohydrate, high-protein dietary interventions. These changes were negatively associated with liver fat deposition as demonstrated through liver magnetic resonance imaging in obese patients with steatotic liver disease [236]. A recent study also reported an increase in *Ruminococcus bromii* abundance after one month of KETO adherence in obese patients, accompanied by a reduction in LPS-producing gut bacteria [237]. In murine models, similar adherence to KETO for a comparable duration resulted in reduced oxidative stress and lower levels of pro-inflammatory cytokines such as TNF-α and IL-6 [273]. Notably, the euglycemic KETO treatment group exhibited a fivefold decrease in hepatic expression of NF-κβ compared to control diets [273]. Since hepatic NF-κβ activation is a major driver of liver steatosis, chronic activation of this pathway promotes de novo lipogenesis, cholesterol synthesis, and MASLD severity [68]. Further, findings from Guevara-Cruz et al. showed that KETO adherence and intermittent fasting correlated with improved mitochondrial function in monocytes, reducing their dependence on glycolysis [237]. Mitochondrial function is an important marker of both metabolic and inflammatory status as mitochondria serve as an important regulator of energy balance and fat accumulation [274].

Another mechanism that may link KETO to liver fat deposition is the microbiota-mediated bile acid metabolism, as this dietary intervention has been shown to significantly influence the composition of BSH-active bacterial phyla, including Bacteroidetes and Actinobacteria [238]. BSH activity can contribute to weight loss through up-regulating both TGR5 and FXR signaling pathways and the associated increased metabolic rate in brown fat tissue [275]. Recent studies have suggested that KETO adherence promotes increased circulating levels of secondary bile acids taurodeoxycholic acid (TDCA) and tauroursodeoxycholic acid (TUDCA), promoting weight loss and blood glucose regulation [276,277]. Specifically, TUDCA inhibits FXR expression and fatty acid transport protein 5 (FATP5), reducing hepatic fatty acid absorption and lipid accumulation [277].

While KETO offers significant metabolic benefits, its low caloric and high fat composition poses risks, particularly with long-term adherence. It is important to note that the studies described above range from 6 days to 8 weeks and therefore do not provide sufficient evidence of the long-term effects of KETO adherence and any potential adverse effects that may be associated with this dietary pattern. Pure calorie-restrictive diets may lead to changes in serum bile acid profiles and gut microbiota [278], with associated down-regulation of uncoupling protein 1 (UCP1), an important regulator of energy expenditure [279]; weight rebound [278]; and liver steatosis [280]. The high-fat nature of this diet can paradoxically exacerbate liver damage over the long term by increasing fatty acid influx, causing nutrient deficiencies, kidney stress, and increases in LDL levels [281,282]. While data on humans are scarce, studies in murine models have shown that 12 weeks of KETO adherence can contribute to liver fat accumulation [283]. In these mice, SREBP1 was not up-regulated, indicating that de novo lipogenesis was not the driving factor of hepatic steatosis but rather an increase in saturated fatty acid uptake, which was found to induce hepatic inflammation and elevate LDL concentrations [283]. Therefore, the quality of fats consumed becomes critical to optimizing the benefits of KETO while minimizing its risks. These include fats from nutrient-dense foods such as avocados, olives, nuts, and fatty fish [284]. As such, KETO should be carefully managed under the guidance of a dietician or healthcare professional with regular monitoring of serum ketones, lipids, and liver function tests, especially during prolonged adherence.

### 4.3. Intermittent Fasting, Gut Microbiota, and MASLD

Intermittent fasting (IF) is a dietary practice characterized by alternating periods of fasting and eating designed to extend the time between calorie intake and encourage fat breakdown [285]. Popular methods of IF include alternate-day fasting, 5:2 fasting regimen, and time-restricted eating [285]. This dietary approach has been associated with numerous health benefits including low-grade inflammation, improved gut health, better glucose regulation, optimized lipid profiles, and effective weight loss [286,287]. A recent meta-analysis of 14 studies involving 840 participants with MASLD undergoing IF demonstrated significant improvements in metabolic end points including reductions in liver enzymes, body weight, waist to hip ratio, hepatic steatosis, and liver stiffness as measured by elastography [239]. Interestingly, various IF regimens have shown different, but generally positive, outcomes in managing MASLD [288]. Specifically, the 5:2 fasting regimen and time-restricted eating significantly improved liver stiffness and steatosis, while alternate-day fasting had a more pronounced impact on reducing fat mass and body weight [288].

Physiologically, intermittent fasting is intricately linked with signaling pathways and metabolites that regulate hepatic fat deposition [289]. For example, peroxisome-proliferator-active receptor α (PPARα) and phosphoenolpyruvate carboxykinase 1 (PCK1), whose enzymatic activity are reduced in individuals with MASLD, are up-regulated significantly with adherence to an IF regimen [240]. Studies have shown that PPARα expression increases after 6 h of time-restricted feeding, contributing to improved lipolysis, fat digestion, and absorption [241]. The effects of PPARα following intermittent fasting are shown to ameliorate the progression of steatotic liver disease to fibrosis and even hepatic cell carcinogenesis [240]. These beneficial effects were absent in mice models with knockdown of PPARα and PCK1 genes. Interestingly, PPARα activation has been shown to increase microbial diversity, restore intestinal barrier integrity, and mitigate steatosis [290]. Conversely, knockdown of PPARα induces gut dysbiosis [291], while reduced microbial diversity disrupts PPARα activity, promoting inflammation [292]. These findings highlight a symbiotic relationship between PPARα activity and gut microbiota through the gut–liver axis in steatotic liver disease models [293].

Adherence to IF has been shown to attenuate MASLD by enhancing autophagy [294], a critical cellular process that recycles cellular components, particularly under conditions of nutrient deprivation and metabolic stress [295]. In steatotic liver disease, autophagy protects against disease progression by clearing excess lipid droplets from hepatocytes, reducing oxidative stress, inflammation, and fibrosis [295]. However, these autophagic processes, are often impaired in MASLD, exacerbating disease progression [296]. IF up-regulates autophagy–lysosome activity in Kupffer cells, counteracting the impaired autophagic flux associated with steatotic liver disease [294]. Microbiota are potent modulators of hepatic autophagy through metabolites like secondary bile acids and SCFA [297]. Activation of the bile acid receptors TGR5 and FXR positively regulates autophagic processes. Consequently, microbial conversion of primary to secondary bile acids can facilitate lipid clearance from hepatocytes via receptor agonism [298]. Similarly, increased relative abundances of butyrate and propionate have been shown to induce autophagy–lysosome activity by up-regulating uncoupling protein 2 expression and hepatic AMPK, which recruit autophagy-related proteins [299].

In addition to its effects on autophagy, IF-induced microbial changes contribute to weight loss and browning of white adipose tissue, improving metabolic parameters in steatotic liver disease [300]. Interestingly, microbiota-depleted models exhibited resistance to these beneficial effects [300], highlighting the pivotal role of gut microbiota in mediating IF outcomes related to MASLD [243]. A meta-analysis of eight studies evaluating the effect of IF on microbiota in individuals with metabolic disease demonstrated significant improvements in microbial richness and α-diversity, although the specific microbial subtypes within these studies showed heterogeneity [242]. Studies have shown significant enrichment of *Parabacteroides distasonis* and *Bacteroides thetaiotaomicron* after a three week IF regimen [243]. *Parabacteroides distasonis* has been linked to the amelioration of MASLD progression through the interaction with dietary inulin to produce hepatoprotective odd-chain fatty acids [301]. These mechanisms reduced serum LPS levels and hepatic pro-inflammatory cytokine expression murine models of MASLD [301]. Additionally, *Parabacteroides distasonis* is correlated with increased BSH-activity, inhibition of FXR signaling, and decreased accumulation of toxic bile acids, notably TCDCA in liver cells [302]. TCDCA is known to produce mitochondrial permeability and increase pyroptosis in hepatocytes, thereby contributing to hepatic fibrosis [102]. As such, MASLD progression may be ameliorated by *Parabacteroides distasonis* via the enhancement of bile acid secretion [302]. Additionally, *Parabacteroides distasonis* has been shown to improve insulin sensitivity through strengthening the gut barrier via activation of intestinal G-coupled protein receptors in the intestinal tract [303]. Similarly, *Bacteroides thetaiotaomicron* exhibits numerous beneficial effects on liver fat accumulation and metabolic processes [304]. In murine models, treatment with *Bacteroides thetaiotaomicron* reduced hepatic steatosis and LPS translocation, up-regulated glucagon-like peptide 1, down-regulated fibroblast growth factor 15, and improved lipid metabolism and fatty acid oxidation [304]. Additionally, this bacterium alleviated metabolic dysfunction by generating hepatic metabolites that counteract oxidative stress [305]. Therefore, by increasing the abundance of these key microbes that play pivotal roles in MASLD pathophysiology, intermittent fasting may significantly improve steatotic liver disease outcomes through microbiota-derived mechanisms.

### 4.4. High Fiber Diet, Gut Microbiota, and MASLD

Dietary fiber has demonstrated significant metabolic benefits, particularly in the context of steatotic liver disease, largely due to its role in modulating gut microbiota [306]. High fiber intake, often a component of the MedDiet, serves as an important prebiotic, supporting the growth and survival of beneficial microbial species, conferring improvements in insulin resistance, systemic inflammation, weight loss, and hepatic fat accumulation [307,308]. Fiber types include soluble and insoluble, with soluble fibers found in oats, legumes, and fruits, which dissolve in water and are fermented by bacterial enzymes within the large intestine, yielding metabolically active compounds like SCFA [309]. Insoluble fibers, on the other hand, are found in grains, vegetables, and nuts; however, they are not generally metabolized by microbial enzymes but still contribute to overall gut health [310]. Fiber consumption is inversely associated with hepatic fibrosis and steatotic liver disease. For instance, individuals in the highest tertile of fiber consumption had significantly reduced odds (OR = 0.81) of developing clinically significant hepatic fibrosis, as assessed by transient elastography [244]. Another study demonstrated that individuals in the highest quartile of fiber intake had the lowest odds (OR = 0.12) of developing steatotic liver disease, compared to higher-risk quartiles with lower fiber consumption [245].

The fermentation of soluble fibers in the gastrointestinal tract influences the microbial environment, playing a crucial role in MASLD pathogenesis and progression [311]. In a 60-day study, MASLD patients consuming 6–12 g of fiber daily exhibited significantly enhanced microbial diversity and increased fecal SCFA concentrations, particularly butyrate and acetate [246]. For example, dietary oat β-glucan, a beneficial prebiotic fiber, dampened MASLD-related inflammation and prevented disease progression [247]. The mechanisms by which fibrotic progression was diminished included reductions in monocyte-derived macrophage infiltration into hepatocytes, TLR ligand translocation, and suppression of fibroinflammatory gene expression. These effects were mediated by gut microbial changes following oat β-glucan intervention as antibiotic depletion abolished the observed benefits, highlighting the indispensable role of microbial taxa such as *Ruminococcus* and SCFA-producing *Lactobacillus* [247]. Increased SCFA concentrations following β-glucan consumption may activate the AMPK signaling pathway, inhibiting de novo lipogenesis and reducing hepatic liver lipid deposition [312]. Additionally, oat bran consumption has been shown to ameliorate hepatic inflammation through alterations in gut metabolites, such as bile acids and tryptophan [313]. Tryptophan, when diverted to the indole pathway by microbial activity, produces indole-3-acetic acid and indole-3 propionic acid [314]. In MASLD, the diversion of tryptophan to the indole pathway is diminished, an effect rescued after dietary oat intake [313]. Indole-3-acetic acid and indole-3 propionic acid may ameliorate hepatic steatosis and inflammation via inhibition of NF-κβ signaling, reduction in endotoxin levels and inactivation of macrophages [315]. Therapeutic supplementation with these metabolites has shown promising potential in MASLD treatment [315].

Inulin, a dietary fiber found in fruits and grains, also exerts beneficial effects in MASLD. It enhances gut barrier integrity by up-regulating tight junction protein expression, an effect mediated by increased abundances of SCFA-producing genera such as *Bifidobacterium*, *Phascolarctobacterium*, and *Blautia* [248]. Inulin supplementation also modulates key lipogenic enzymes such as PPARα, ChREBP, and stearoyl-CoA desaturase-1. Additionally, inulin increases relative concentrations of *Akkermansia* by fivefold [249]. *Akkermansia*, has emerged as an important player in the gut–liver axis [316]. Its treatment has been associated with increased mitochondrial oxidation, improved bile acid metabolism, and reduced metabolic stress [316]. Specifically, *Akkermansia* enhances the production of hepatoprotective metabolites such as L-aspartate, which prevents lipid peroxidation, improves hepatic microcirculation, and exerts anti-inflammatory effects [317]. Additionally, *Akkermansia* decreases IL-6 levels and reduces triglyceride synthesis, mitigating lipid peroxidation in steatotic liver disease models [318]. Through the modulation of microbial composition and metabolite production, high-fiber diets confer significant protective effects against hepatic fat accumulation, inflammation, and fibrosis in MASLD. Soluble fibers enhance SCFA production, improve intestinal barrier integrity, and regulate key hepatic pathways, highlighting the therapeutic potential of fiber as a dietary intervention for MASLD.

## 5. Conclusions and Perspective

This review addresses critical gaps in understanding by elucidating specific microbial roles, metabolite pathways, and host interactions involved in the relationship between diet, gut microbes, and MASLD. As presented, substantial evidence supports the role of the gut microbiota as a key mediator of the beneficial or harmful effects of dietary patterns on steatotic liver disease. Unfavorable dietary patterns with high cholesterol, high fructose, high saturated fat, and low protein are associated with an increased relative abundance of dysbiosis-inducing bacterial genera such as *Enterococcus* and *Escherichia*, which are linked with poor MASLD outcomes. These dietary patterns up-regulate inflammation through the LPS/TLR4/NF-κβ pathways, increase barrier permeability, disrupt bile acid metabolism, and elevate the expression of lipogenic enzymes, collectively driving hepatic fat accumulation. In contrast, favorable dietary patterns such as the MedDiet, KETO, IF, and high fiber diets exert positive effects on microbial composition and enhance metabolite profiles, improving inflammatory, oxidative, lipogenic, and related metabolic pathways. These effects mitigate fatty liver accumulation and contribute to beneficial MASLD outcomes. Key microbial taxa driving these benefits include *Rumminococcacae*, with genera *Ruminococcus*, *Akkermansia*, *Lactobacillus*, *Blautia*, *Faecalibacterium*, *Bacteroides*, and *Prevotella*, which are associated with enhanced metabolic parameters, strengthened gut barrier integrity, and reduced systemic inflammation. Generally, the MedDiet and high-fiber diets have been shown to be highly safe and beneficial across a wide range of metabolic conditions beyond MASLD, even with long-term adherence [319], with microbiota serving as significant drivers of its health benefits [320]. In contrast, while KETO has demonstrated short-term efficacy in improving MASLD parameters, its long-term adherence requires caution and should be undertaken under professional supervision. Therefore, future research and large-scale clinical trials should focus on assessing long-term safety and efficacy of dietary interventions such as KETO in hepatic steatosis as current evidence on humans remains limited. Additionally, incorporating detailed gut microbial and metabolite analysis in studies involving these dietary interventions will provide deeper insights into the mechanisms by which taxonomical shifts in gut microbiota influence MASLD outcomes. This will advance the development of novel dietary and microbiota-based therapeutic approaches for MASLD.

Overall, while gut microbiota and dietary patterns significantly influence MASLD development and progression, they are only part of a comprehensive treatment approach. Physical activity (e.g., aerobic exercise, resistance training), weight management (e.g., loss and maintenance), and behavioral modifications (e.g., stress management, sleep optimization) are equally essential components of steatotic liver disease management. Therefore, microbiota-targeted dietary intervention should be integrated in conjunction with these lifestyle modifications, when possible, to achieve optimal outcomes for patients with MASLD.

## Figures and Tables

**Table 1:** Effects of dietary patterns on gut microbiota, related metabolites, and MASLD. Abbreviations: MASLD, metabolic-dysfunction associated steatotic liver disease; OR, odds ratio; TC, total cholesterol; kPa, kilopascal; ALT, alanine aminotransferase; WBC, white blood cell; CRP, C-reactive protein; OTU, operational taxonomic units; r, correlation coefficient; LPS, lipopolysaccharides; bsh, bile-salt hydrolase; AST, aspartate aminotransferase; BMI, Body Mass Index; PPARα, peroxisome-proliferator-activated receptor alpha; PCK1, phosphoenolpyruvate carboxykinase 1; FAS, fatty acid synthase; SCFA, short-chain fatty acids; TLR, toll-like receptor.

Dietary Intervention	Study Type/Study Duration/Sample Size	Results/Implications	Reference
Low-glycemic Mediterranean diet (MedDiet)	Retrospective cohort study/2 years/5395 patients	High adherence to the diet was significantly associated with lower rates of MASLD (OR = 0.839) Lowered triglycerides, glucose index, atherosclerosis	[228]
	Meta-analysis/10 studies (mean duration 6 weeks to 1 year)/737 patients	Reduced MASLD severity, TC, liver fibrosis, waist circumference Reduced liver stiffness by 0.42 kPa	[229]
	Meta-analysis/26 studies/3037 patients	Reduced ALT, fatty liver index, and liver stiffness	[230]
	Prospective study/100 patients	High adherence to the diet improved fatty acid profile, inflammatory status, and oxidative stress	[12]
	RCT/5 years/109 patients	Adherence increased *Lachnospiraceae*, *Rumminococcacae*, and *Oscillospiraceae* Genera *Ruminococcus*, *Akkermansia*, *Eubacterium*, *Dialister*, *Alistipes*, and *Clostridium* Reduced severity of liver steatosis	[231]
	RCT/1 year/297 patients	Distinct differences in microbial composition depending on degree of fibrosis (Tertile 1–3) Tertile 3 showed decrease *Enterobacteriaceae* and increase *Sutterella*, *Faecalibacterium*, and *Blautia* in response to MedDiet	[232]
	Meta-analysis/37 articles	Adherence increased microbial diversity and *Faecalibacterium* and *Prevotella* Reduced inflammation, improved glycemic control, lowered fat mass, and hospitalization risk	[233]
Ketogenic diet (KETO)	Experimental study/6 days/10 patients	Short-term KETO decreased intrahepatic triglycerides by 31% and hepatic insulin resistance by 58% 238% increase in portioning of fatty acids towards ketogenesis 50% drop in insulin levels 167% improvement in hepatic mitochondrial redox state	[234]
	Prospective study/8 weeks/87 patients	Decreased WBC count, TC, insulin resistance, and liver fibrosis Reduced low-grade inflammation	[235]
	Pilot study/3 weeks/15 patients	Decreased body weight by 3.6%, liver fat by 65%, and CRP by 19% Decreased *Lachnospira* Increased *Butyricicoccus* and *Blautia* Liver fat was negatively correlated with *Rumminococcacae* (OTU, r = −0.83)	[236]
	RCT/1 month/44 patients	Increased *Phascolarctobacterium faecium* and *Ruminococcus bromii* Decreased LPS-related signaling Improved mitochondrial function and monocyte dependence	[237]
	Comparative study/4 weeks/17 patients	Altered community structure of bsh-active gut microbiota Increased Bacteroidetes and decreased Actinobacteria	[238]
Intermittent fasting (IF)	Meta-analysis/fourteen studies/840 patients	Improved serum ALT, AST, and hepatic steatosis Improved body weight, BMI, and waist-to-hip ratio	[239]
	Experimental study/5:2 IF regimen/murine model	5:2 regimen increased PPARα and PCK1 to lower hepatic triglycerides and hepatic steatosis Decreased inflammation and fibrosis	[240]
	Experimental study/time-restricted feeding for 15 weeks/murine models	Improved expression of FAS and PPARα genes Enrichment of metabolites associated with lipolysis, fat digestion, and absorption Improved MASLD parameters including body fat ratio, AST, TC, blood glucose, and triglycerides	[241]
	Meta-analysis/8 studies/(n = 9 to n = 80 in those 8 studies)	Significant improvements in microbial richness and α-diversity	[242]
	Prospective study/3 weeks/72 patients	5:2 IF regimen enriched *Parabacteroides distasonis* and *Bacteroides thetaiotaomicron * Increased SCFA production Improved metabolic parameters	[243]
High-fiber diet	Cross-sectional study/N/A/1465 patients	Highest tertile of dietary fiber consumers was associated with lower odds of steatotic liver disease (OR = 0.81) and clinically significant fibrosis (OR = 0.81) compared to the lowest tertile	[244]
	Retrospective study/N/A/6613 patients	Dose response effects of increased dietary fiber with reduced steatotic liver disease risk Bottom half of fiber consumers exhibited similar results, though the top quartiles had significantly lower odds of disease onset	[245]
	Experimental study/2 months/26 patients	Increased concentrations of SCFA (acetate and butyrate) Amount of fiber improved phyla diversity in MASLD patients at one month and two months	[246]
	Experimental study/24 weeks/murine model	Expansion of Ruminococcus and *Lactobacillus* Reduced translocation of TLR Reversed inflammatory and fibrotic changes associated with diet-induced MASLD	[247]
	Experimental study/murine models	Increased abundances of SCFA-producing genera *Bifidobacterium*, *Phascolarctobacterium*, and *Blautia* Improved tight junction proteins and fortified intestinal barrier Alleviated hepatic steatosis	[248]
	Experimental study/4 weeks/murine models.	Inulin supplementation increased relative concentrations of *Akkermansia* fivefold Improved hepatic steatosis	[249]
